# Trends in home dialysis use differ among age categories in past two decades: A Dutch registry study

**DOI:** 10.1111/eci.13656

**Published:** 2021-08-02

**Authors:** Anna A. Bonenkamp, Tiny Hoekstra, Marc H. Hemmelder, Anita van Eck van der Sluijs, Alferso C. Abrahams, Frans J. van Ittersum, Brigit C. van Jaarsveld

**Affiliations:** ^1^ Department of Nephrology Amsterdam UMC, Vrije Universiteit Amsterdam, Research institute Amsterdam Cardiovascular Sciences Amsterdam the Netherlands; ^2^ Dutch Renal Registry (RENINE) Nefrovisie Foundation Utrecht the Netherlands; ^3^ Department of Nephrology Medical University Centre Maastricht Maastricht the Netherlands; ^4^ Department of Nephrology and Hypertension University Medical Centre Utrecht Utrecht the Netherlands

**Keywords:** ageing, end‐stage kidney disease, home dialysis, home haemodialysis, peritoneal dialysis, trends over time

## Abstract

**Background:**

Although the number of patients with end‐stage kidney disease is growing, the number of patients who perform dialysis at home has decreased during the past two decades. The aim of this study was to explore time trends in the use of home dialysis in the Netherlands.

**Methods:**

Dialysis episodes of patients who started dialysis treatment were studied using Dutch registry data (RENINE). The uptake of home dialysis between 1997 through 2016 was evaluated in time periods of 5 years. Home dialysis was defined as start with peritoneal dialysis or home haemodialysis, or transfer to either within 2 years of dialysis initiation. All analyses were stratified for age categories. Mixed model logistic regression analysis was used to adjust for clustering at patient level.

**Results:**

A total of 33 340 dialysis episodes in 31 569 patients were evaluated. Mean age at dialysis initiation increased from 62.5 ± 14.0 to 65.5 ± 14.5 years in in‐centre haemodialysis patients, whereas it increased from 51.9 ± 15.1 to 62.5 ± 14.6 years in home dialysis patients. In patients <65 years, the uptake of home dialysis was significantly lower during each 5‐year period compared with the previous period, whereas kidney transplantation occurred more often. In patients ≥65 years, the incidence of home dialysis remained constant, whereas mortality decreased.

**Conclusions:**

In patients <65 years, the overall use of home dialysis declined consistently over the past 20 years. The age of home dialysis patients increased more rapidly than that of in‐centre dialysis patients. These developments have a significant impact on the organization of home dialysis.

## 1 INTRODUCTION

Globally, the number of patients with chronic kidney disease (CKD) and end‐stage kidney disease (ESKD) is continuing to rise.^1^, ^2^ This growth in the prevalence of patients who need kidney replacement therapy (kidney transplantation or dialysis) causes a major economic and logistical burden to the healthcare system.^1^, ^2^ The majority of patients is treated with in‐centre haemodialysis (CHD), while the use of dialysis at home is low.^3^ But home dialysis offers more flexibility and independence, which could improve quality of life.^4^, ^5^ In addition, home dialysis might be more cost‐effective than CHD.^6^

Another important development is global ageing, also resulting in the ageing of the dialysis population. A further contribution to this is that older patients are not often eligible for kidney transplantation. The ageing of the dialysis population might be a reason for the low use of home dialysis modalities.^7^ In the past, home dialysis generally was performed by young, employed patients. However, nowadays young patients are frequently transplanted with kidneys from living donors.^8^

Consequently, in order to increase the use of home dialysis, it would be helpful to gain better understanding of the impact of age on the home dialysis use, for example to reduce the economic burden of a growing patient population. The aim of this study was to explore time trends in the use of home dialysis in the Netherlands. This country had a pronounced decline in home dialysis patients during the last two decades, and it consistently ranks among the countries with the highest rates of kidney transplantations worldwide.^3^, ^9^, ^10^ Therefore, we studied the uptake of home dialysis between 1997 and 2016 in patients commencing dialysis treatment, stratified for age categories.

## 2 METHODS

### 2.1 Study design

Anonymized registry data from the Dutch Renal Registry (RENINE) were used for this multicentre cohort study. RENINE collects treatment data of dialysis patients in all Dutch dialysis units; >95% of all Dutch dialysis patients are registered in RENINE.^11^ Kidney replacement therapies are registered as CHD, peritoneal dialysis (PD), home haemodialysis (HD) or kidney transplantation. Modality and centre transfers are updated regularly. For this analysis, age at start of dialysis treatment, sex, dates of modality transfers, information on recovery of kidney function, kidney transplantation and death were provided. All patients provided informed consent for registration of the data and usage of data for conducting scientific research. Reporting of the study conforms to broad EQUATOR guidelines.^12^, ^13^

### 2.2 Study population

Dialysis episodes of patients who started *maintenance* dialysis treatment between 1‐1‐1997 through 31‐12‐2016 in the Netherlands were included, including dialysis episodes of patients who previously underwent kidney transplantation. Each dialysis episode was followed for 2 years, and the last day of follow‐up was 31‐12‐2018. A patient may have had multiple dialysis episodes during the study period and may thus be included more than once. Dialysis episodes instead of individual patients were chosen because we considered that a dialysis modality choice is made in each new dialysis episode, including episodes of patients with a dialysis history. Dialysis episodes shorter than 90 days were excluded. In addition, dialysis episodes of patients <20 years of age were excluded, since paediatric care is different from adult patient policy and this patient population is small.

### 2.3 Study outcomes

Primary outcome was start of home dialysis, that is PD and home HD. Both home dialysis at the beginning of the dialysis episode and a transfer to PD or home HD within 2 years of dialysis initiation were included. Subsequent switches after the start of home dialysis were ignored. A complete list of registry codes used to define study outcomes is provided in Appendix S1.

A relatively long transfer period of 2 years was chosen to also include *home HD* patients; in this registry study, the median time to HHD was 16 months [IQR 9‐28] while the median time of transfer to PD was 4 months [IQR 2‐12]. As in literature a shorter transfer period is more common ^14^, start of home dialysis within 12 months of dialysis initiation was also evaluated as a sensitivity analysis.

As secondary outcome, start of PD and start of home HD were analysed separately.

### 2.4 Statistical analysis

The age of incident patients was reported as mean with standard deviation (SD) and sex of incident patients as proportions.

Logistic regression was used to assess the uptake of home dialysis between 1997 through 2016. Calendar time at dialysis initiation was equally divided into 5‐year periods: 1997‐2001, 2002‐2006, 2007‐2011 and 2012‐2016. The period 2002 to 2006 was set as reference category. During this period, the incidence of CHD in the Netherlands peaked after opening of standalone dialysis centres following a governmental decision to allow dialysis treatment in satellite and independent centres.^15^, ^16^ Follow‐up time for each episode was maximum 2 years and censoring occurred at recovery of kidney function, kidney transplantation or death (for corresponding codes, see Appendix S1). A logistic mixed model analysis was performed to adjust for clustering of dialysis episodes at a patient level. This model was additionally adjusted for sex, dialysis vintage and transplantation history. Due to the interaction of age with the different time periods, analyses were stratified for the following age categories: 20‐44 years, 45‐64 years, 65‐74 years or ≥75 years.^17^

A competing risk model was used to estimate the cumulative incidence function (CIF) for start of home dialysis in incident patients with recovery of kidney function, kidney transplantations and all‐cause mortality as competing events.^18^ The 2‐year cumulative incidence is the proportion of the study population, that is incident dialysis patients, who develop the outcome of interest during this time before the occurrence of a competing event. Subsequently, CIFs were estimated for kidney transplantations and all‐cause mortality. In these analyses, the other three outcomes were treated as competing events. The three curves were plotted simultaneously. The curve for all‐cause mortality was plotted as 1 minus CIF.

To further explore the robustness of results, three sensitivity analyses were conducted as follows: (i) home dialysis was defined as start with home dialysis, or transfer to home dialysis within the *first* year after start dialysis—instead of within 2 years; (ii) only the first dialysis episode of patients was analysed, analysing patients instead of dialysis episodes and using logistic regression instead of mixed model logistic regression analysis; and (iii) only episodes of patients who were still treated with dialysis after 2 years were analysed. All incident dialysis episodes followed by recovery of kidney function (n = 771), kidney transplant (n = 4118) or death (n = 7786) within 2 years were excluded, irrespective of dialysis treatment modality.

Overall, a *P*‐value of <.05 was considered statistically significant. All analyses were performed using SPSS Statistics 25 (IBM) or STATA 14.

## 3 RESULTS

**FIGURE 1:**
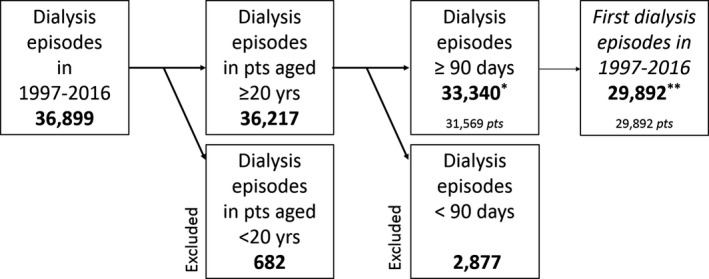
Flow chart of the study. *Main analysis. **Second sensitivity analysis

A total of 33 340 chronic dialysis episodes between 1997 and 2016 fulfilled our inclusion criteria; these episodes belonged to 31 569 adult patients (Figure 1). Table 1 shows the characteristics of dialysis episodes and incident patients included in the study. Both the total number of dialysis episodes as the total number of incident patients increased from 1997 to 2016, whereas the total number of home dialysis episodes decreased (from 3037 to 2390). The total number of home HD was low, yet increased (from 67 to 253). The increase in the total number of incident patients was attributable to the increase in elderly patients: the number of patients aged ≥65 years increased from 2921 to 4889, whereas the number of patients aged 20‐44 years decreased from 1133 to 747.

**TABLE 1:** Characteristics of dialysis episodes and incident patients of the study population, by time period

	1997‐2001	2002‐2006	2007‐2011	2012‐2016
Dialysis episodes				
Total number of dialysis episodes	7230	8107	9004	8999
Total number of home dialysis episodes^a^	3037	2668	2535	2390
Total number of PD episodes^a^	2980	2580	2412	2155
Total number of home HD episodes^a^, ^b^	67	98	131	253
Incident patients				
Total number of incident patients	6496	7329	8047	8020
Aged 20‐44 y	1133	1025	863	747
Aged 45‐64 y	2442	2480	2536	2384
Aged 65‐74 y	1881	2132	2236	2439
Aged ≥75 y	1040	1692	2412	2450
Mean age at start dialysis (y ± SD)	59.6 ± 15.0	62.5 ± 14.8	64.9 ± 14.5	65.6 ± 14.1
Male (%)	3909 (60)	4487 (61)	5002 (62)	5009 (62)

^a^ Within 2 y of dialysis initiation.

^b^ 46 home haemodialysis episodes were preceded by PD treatment.

**FIGURE 2:**
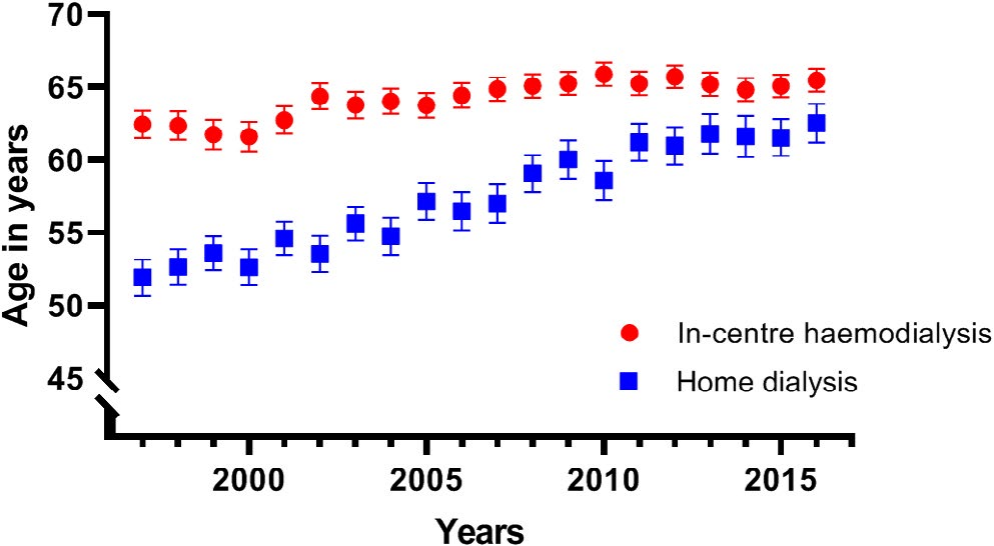
Mean age at start of a dialysis episode between 1997 and 2016. Mean age with confidence intervals. Each dot represents a year from 1997 to 2016

In Figure 2, the mean age in years at the start of a dialysis episode between 1997 and 2016 is shown. The age of home dialysis patients increased from 51.9 ± 15.1 to 62.5 ± 14.6 years during this period, while the age of CHD patients increased from 62.5 ± 14.0 to 65.5 ± 14.5 years.

### 3.1 Time trends in uptake of home dialysis

Table 2 shows the results of the logistic regression assessing the uptake of home dialysis in each time period for the four age categories, using 2002‐2006 as the reference period since governmental policies introduced around this period incentivized the growth of dialysis centres.^15^, ^16^ Table 2A shows the uptake of home dialysis within 2 years of dialysis initiation, and Table 2B shows the start of home dialysis within 1 year of dialysis initiation. During 1997‐2001, for all age categories the uptake of home dialysis was significantly higher compared with the reference period (adjusted odds ratios (OR) ranging from 1.30 to 2.17; Table 2A). In the youngest two age categories, that is dialysis episodes of patients <65 years, the uptake of home dialysis was significantly lower in time periods 2007‐2011 and 2012‐2016 than for the period 2002‐2006 (adjusted ORs ranging from 0.36 to 0.63). Each time period of 5 years was associated with a significantly lower uptake of home dialysis compared with the previous period in these age categories. In the 65‐ to 74‐year category, adjusted ORs in the time periods 2007‐2011 and 2012‐2016 were not significantly different from the reference period. In patients aged ≥75 years, the two most recent time periods were associated with a higher uptake of home dialysis (adjusted ORs 1.21 and 1.52 resp.) compared with the reference period. As findings were similar for the analyses with, respectively, a 2‐year transfer period and a 1‐year transfer period, all further analyses were performed with a transfer period of 2 years to allow for the longer transfer time of home HD.

**TABLE 2:** Uptake of home dialysis in 2 y of dialysis initiation (A) and in the *first year* of dialysis initiation (B; n = 33 340), by time period and age category

A		Time period			
		1997‐2001 OR (95% CI)	2002‐2006 ^a^	2007‐2011 OR (95% CI)	2012‐2016 OR (95% CI)
Age 20‐44	Unadjusted	2.24 (1.70‐2.94)	1.0	0.52 (0.40‐0.69)	0.34 (0.25‐0.46)
	Adjusted^b^	2.17 (1.66‐2.84)	1.0	0.54 (0.42‐0.72)	0.36 (0.27‐0.49)
Age 45‐64	Unadjusted	1.61 (1.35‐1.92)	1.0	0.62 (0.52‐0.74)	0.44 (0.36‐0.53)
	Adjusted^b^	1.60 (1.34‐1.91)	1.0	0.63 (0.53‐0.75)	0.43 (0.36‐0.53)
Age 65‐74	Unadjusted	1.30 (1.14‐1.49)	1.0	0.95 (0.84‐1.09)	0.98 (0.86‐1.11)
	Adjusted^b^	1.30 (1.14‐1.49)	1.0	0.95 (0.84‐1.09)	0.98 (0.86‐1.12)
Age above 75	Unadjusted	1.35 (1.10‐1.66)	1.0	1.22 (1.03‐1.45)	1.54 (1‐1.81)
	Adjusted^b^	1.35 (1.10‐1.67)	1.0	1.21 (1.02‐1.44)	1.52 (1.29‐1.80)

B		Time period			
		1997‐2001 OR (95% CI)	2002‐2006 ^a^	2007‐2011 OR (95% CI)	2012‐2016 OR (95% CI)
Age 20‐44	Unadjusted	2.44 (1.82‐3.27)	1.0	0.49 (0.37‐0.67)	0.32 (0.23‐0.44)
	Adjusted^b^	2.35 (1.77‐3.13)	1.0	0.53 (0.40‐0.70)	0.35 (0.26‐0.48)
Age 45‐64	Unadjusted	1.63 (1.37‐1.95)	1.0	0.60 (0.50‐0.71)	0.41 (0.34‐0.50)
	Adjusted^b^	1.62 (1.35‐1.95)	1.0	0.60 (0.50‐0.72)	0.41 (0.34‐0.51)
Age 65‐74	Unadjusted	1.28 (1.12‐1.47)	1.0	0.94 (0.82‐1.08)	0.93 (0.82‐1.06)
	Adjusted^b^	1.28 (1.12‐1.47)	1.0	0.94 (0.83‐1.07)	0.94 (0.82‐1.07)
Age above 75	Unadjusted	1.37 (1.11‐1.68)	1.0	1.21 (1.02‐1.44)	1.50 (1.27‐1.78)
	Adjusted^b^	1.36 (1.11‐1.68)	1.0	1.20 (1.01‐1.43)	1.49 (1.25‐1.76)

^a^ Time period 2002‐2006 was regarded as reference period.

^b^ Adjusted for sex, dialysis vintage and transplantation history.

### 3.2 Time trends in uptake of PD or home HD

The uptake of PD was quite similar to the overall uptake of home dialysis (Table 3). However in the 65‐ to 74‐year category, the last time period was associated with a borderline significant lower uptake of PD (adjusted OR 0.89, 95% CI 0.78‐1.01).

**TABLE 3:** Uptake of peritoneal dialysis within 2 y of dialysis initiation (n = 33 340), by time period and age category

		Time period			
		1997‐2001 OR (95% CI)	2002‐2006^a^	2007‐2011 OR (95% CI)	2012‐2016 OR (95% CI)
Age 20‐44 y	Unadjusted	2.41 (1.81‐3.22)	1.0	0.46 (0.34‐0.62)	0.26 (0.19‐0.37)
	Adjusted^b^	2.33 (1.75‐3.11)	1.0	0.49 (0.36‐0.66)	0.28 (0.20‐0.39)
Age 45‐64 y	Unadjusted	1.68 (1.40‐2.01)	1.0	0.60 (0.50‐0.72)	0.38 (0.31‐0.46)
	Adjusted^b^	1.67 (1.39‐2.00)	1.0	0.60 (0.50‐0.72)	0.37 (0.30‐0.46)
Age 65‐74 y	Unadjusted	1.31 (1.15‐1.50)	1.0	0.93 (0.81‐1.06)	0.88 (0.78‐1.01)
	Adjusted^b^	1.31 (1.15‐1.50)	1.0	0.93 (0.82‐1.07)	0.89 (0.78‐1.01)
Age above 75 y	Unadjusted	1.36 (1.10‐1.67)	1.0	1.22 (1.03‐1.45)	1.40 (1.18‐1.66)
	Adjusted^b^	1.36 (1.10‐1.67)	1.0	1.21 (1.02‐1.44)	1.39 (1.17‐1.64)

^a^ Time period 2002‐2006 was regarded as reference period.

^b^ Adjusted for sex, dialysis vintage and transplantation history.

After correction for sex, age, dialysis vintage and transplantation history, the home HD use increased for each time period (Table S1). The last time period had an adjusted OR of 3.57 (2.59‐4.92). As the number of home HD episodes was too low, no stratification for age categories was performed.

### 3.3 Time trends in the incidence of home dialysis

**FIGURE 3:**
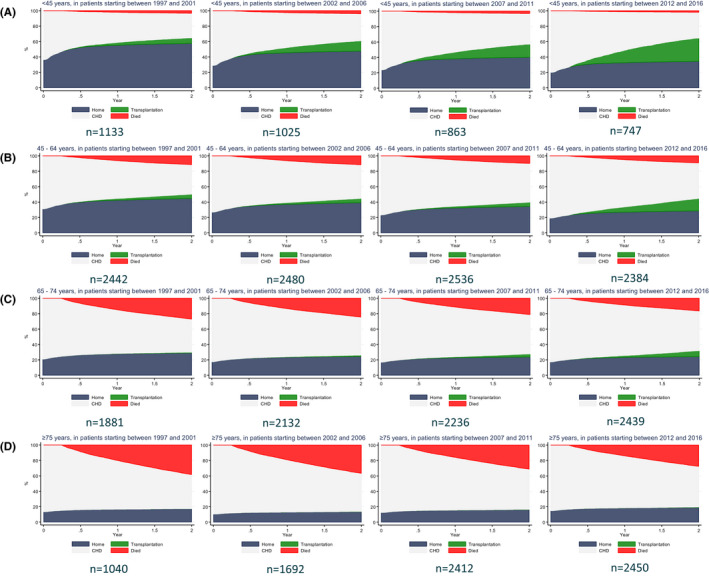
Cumulative 2‐y incidences of home dialysis, kidney transplantation, CHD and death in incident patients. A, 20‐44 y; B, 45‐64 y; C, 65‐74 y; D, ≥75 y [Correction added on 23 September 2021 after first online publication: Figure 3 was corrected in this version]

Figure 3 shows the results of a competing risk approach modelling the cumulative incidences for start of home dialysis and those for kidney transplantation and death following CHD within 2 years after dialysis initiation, categorized by time period and age group. Figure 3A shows that the 2‐year incidence of home dialysis for patients aged 20‐44 years decreased in subsequent time periods from 58% to 34%. Figure 3B shows that the 2‐year incidence for patients aged 45‐64 years also decreased from 45% to 29%. In patients aged 65‐74 years, the 2‐year incidence of home dialysis was 29% in time period 1997‐2001 and remained 24% during the other time periods (Figure 3C). In patients aged ≥75 years, the 2‐year incidence of home dialysis was low: 17% in the first period, 14% in the second period, 16% in the third period and 19% in the last time period (Figure 3D).

In the youngest age groups, the 2‐year incidence of kidney transplantation while on CHD increased considerably, from 7% to 30% in patients aged 20‐44 years and from 5% to 16% in patients aged 45‐64 years. In patients aged 65‐74 years, the incidence of kidney transplantation was 8% during the last time period, and in patients aged >75 years, this incidence was almost nihil. In the time period 1997‐2001, the 2‐year incidence of home dialysis and kidney transplantations combined was 65% for patients aged 20‐44 years, which was comparable with the combined incidence in the time period 2012‐2016 in this age category. In patients aged 45‐64 years, the combined 2‐year incidence was 50% in 1997‐2001 and 45% in 2012‐2016. In patients aged 65‐74 years, the 2‐year incidences were 30% and 32% respectively.

The 2‐year incidence of mortality on CHD decreased over the time periods for all age categories (Figure 3A‐D). This phenomenon was most pronounced in older patients: the incidence of mortality decreased from 27% to 16% in patients aged 65‐74 years and from 38% to 27% in patients aged ≥75 years. In addition, the proportion of patients that stayed on CHD increased from 43% to 52% in patients aged 65‐74 years and from 44% to 53% in patients aged ≥75 years.

### 3.4 Sensitivity analyses

Table S2 shows the results of the sensitivity analysis of only first dialysis episodes between 1997 and 2016. A total of 29 892 patients were analysed. The uptake of home dialysis was still significantly lower in the last two time periods for patients <65 years old, yet ORs tended to be higher if compared to the original analysis. The OR for home dialysis uptake in period 2012‐2016 was 0.54 (95% CI 0.45‐0.66) in patients aged 20‐44 years and 0.59 (95% CI 0.52‐0.66) in patients aged 45‐64 years. In the third sensitivity analysis, only episodes of patients that were still on dialysis 2 years after dialysis initiation were evaluated, excluding episodes that ended with recovery of kidney function, kidney transplantation or death. A home dialysis episode was still defined according to the definition used in the original analysis, that is start or transfer to home dialysis within 2 years of dialysis initiation. The results of this analysis were similar to the results from the original analysis, except that in dialysis episodes of patients ≥75 years of age the uptake of home dialysis during the first time period was no longer significantly higher compared with the reference period (Table S3).

## 4 DISCUSSION

In this large cohort of Dutch patients, the home dialysis use in patients aged <65 years declined over the time periods from 1997 to 2016. In these younger patients, a considerable increase in the number of kidney transplants was seen. In contrast, the older population showed a constant home dialysis use over time for the patients 65‐74 years of age and a significant increase for the patients above 75 years of age. As a result, the home dialysis population aged remarkably. In both elderly age groups, kidney transplantation was negligible, but a clear decrease in mortality was found in elderly patients starting dialysis. Most of the elderly patients remained on CHD over time. The predominantly used home dialysis treatment in this cohort was PD. Although numbers are low, over time the home HD use increased.

Multiple factors possibly influenced the changes in the uptake of home dialysis. In the first time period, 1997‐2001, the uptake of PD was quite high, in part explained by a shortage of CHD facilities. After a change in legislation regarding initiating a dialysis centre by the Dutch government in 1999, this capacity problem was resolved. Consequently, many new dialysis centres appeared and an increase in patients starting CHD was observed in 2002.^15^, ^16^ Apparently, such a policy change can have a major influence on the choice of dialysis modality within a population, as has also been reported in North America.^19^, ^20^ In contrast, in Australia and China governmental initiatives to promote home dialysis have resulted in a stabilization or even an increase in the prevalence of home dialysis patients.^14^, ^21^ In such large countries with extensive rural areas and great distance to the nearest dialysis centre, home dialysis may be a favoured treatment.^21^ Indeed, in China 20% of the total dialysis population is treated with PD, while in Australia 25% is treated with home dialysis.^21^, ^22^ The small country of Hong Kong has even the highest percentage of home dialysis throughout the world, as 76% of dialysis patients are treated with PD, due to a three‐decade PD‐first policy adopted due to its cost‐effectiveness.^23^ Another country in which policy changes had a marked effect is the USA. In 2018, this country had a total of 12% of dialysis patients on a home‐based therapy compared with 9% prior to differences in reimbursement.^24^ Main reasons for changing the reimbursement were rising healthcare costs and improving healthcare efficiency.^21^, ^25^ In Europe, the proportion varies from 7% in Greece to 30% in Scandinavian countries such as Finland.^3^ In the latter, a home first policy was adopted, partly due to a capacity problem but more importantly to provide individualized dialysis treatment which may be best achieved at home.^26^ Overall, practices in these countries suggest that governmental policies to promote home dialysis are important and can have a large impact on uptake of home dialysis. In the present analysis, the initial decrease in home dialysis came to a halt in the time periods following 2002‐2006 in the elderly patient groups, possibly due to dedication and initiatives of both nephrologists and nurses who stimulated home dialysis in these patients.

The average home dialysis patient aged significantly over a period of 20 years. First, this can be explained by the ageing of the total dialysis population since more elderly patients started dialysis. Second, this can be explained by less younger patients starting home dialysis, since these patients are more often transplanted. This suggestion is supported by the fact that the CHD population has aged less than the home dialysis population and that we observed a clear increase in kidney transplantations after CHD initiation in patients under <75 years of age. This trend is in agreement with European data.^27^ One could claim that ‘the home dialysis patient of 20 years ago obtains a kidney transplant at present’. Indeed, in the younger population the combined 2‐year incidence of transplantation and home dialysis remained more or less the same over a period of 20 years.

In addition, a decrease in the 2‐year incidence in mortality on CHD was found from 1997 to 2016, most pronounced in patients above 65 years of age. This is consistent with a recent study of the ERA‐EDTA, contributing a 10‐year reduction in mortality not solely to a better survival in the general population, but also to improvements in dialysis care.^28^ As a consequence of the decreased mortality rates, more elderly patients are on long‐term maintenance dialysis. The majority of these patients is treated with CHD; the 2‐year incidence of home dialysis in most elderly patients (≥75 years) increased only slightly from 17% to 19%. The low incidence of home dialysis in the elderly patients might be explained by the notion that elderly patients are too frail to be treated with home dialysis.^7^ However, the greater proportion of patients staying on CHD over time could also suggest that more elderly patients would be able to start home dialysis if sufficiently assisted. Nevertheless, the ageing of the dialysis population will have implications for the organization of pre‐dialysis education and home dialysis, as older patients may require additional support.

Over the past 15 years, several international initiatives were introduced to promote home dialysis, especially in elderly patients.^29^ These initiatives include training of community‐based home care workers to perform dialysis tasks at the patient's home, prolonging training time for the elderly patient and updating educating programmes to enhance informed decision‐making.^30^, ^31^, ^32^ Although we observed a 50% higher uptake of home dialysis in patients above 75 years of age, the overall use in these elderly patients remained low: the proportion increased from 14% in the reference period, that is 2002‐2006, to 19% in the most recent time period. It should also be noted that the 2‐year incidence for home dialysis was 17% in the time period prior to the governmental legislation. Thus, the abovementioned initiatives possibly helped to revive home dialysis after the governmental decision. In other countries, a higher proportion of elderly patients is treated with home dialysis. Especially in Australia and New Zealand, this proportion is quite high, 24% and 47%, respectively, suggesting that it is possible for many elderly patients to perform home dialysis.^33^ Incorporating more initiatives to promote home dialysis may allow more elderly patients to start home dialysis in the future.

The growing number of, especially elderly, dialysis patients puts pressure on healthcare expenses worldwide, since dialysis is an expensive treatment.^1^, ^2^ Home dialysis might be a mean of relieving this financial burden, since especially continuous ambulatory PD is supposed to be more cost‐effective.^6^ Moreover, elderly patients may as well benefit from home dialysis: they might obtain better quality of life and might be more satisfied with assisted PD than with CHD.^4^, ^34^ However, home dialysis in elderly patients emphasizes the need for adaptation in organization of home dialysis care, yet total expenses, including those for home care workers, remains unknown. The results presented in this study have implications for further research and underscore the need of cost‐effectiveness studies in elderly patients.

The results of our study remained robust in three different sensitivity analyses, a strength of this study. Other strengths of this study include its large sample size and the inclusion of dialysis episodes of nearly all chronic dialysis patients in the Netherlands over 20 years. This enabled us to explore in detail the various shifts in kidney replacement therapy and in competing events, that is kidney transplantation and mortality, over time. However, registry data are also a limitation to this study. Not all potentially relevant confounders are registered in the registry; we were, for example, unable to explore the effect of a pre‐dialysis education programme.^35^ Other patient‐specific characteristics that are known to influence dialysis modality choice, such as comorbidities and acute start of dialysis, could also have changed the main results since these demographic characteristics have supposedly changed over time in the home dialysis population.^14^, ^30^, ^36^ We evaluated shifts in kidney replacement therapy *after* dialysis initiation; the effect of pre‐emptive kidney transplants is not evaluated in the present analysis.^11^ Furthermore, not necessarily a limitation but noteworthy nevertheless, we presented the 2‐year incidences of kidney transplantation and mortality for incident patients initiating CHD, not the kidney transplantation and mortality incidences for patients that initiated treatment with home dialysis.

## 5 CONCLUSIONS

From 1997 to 2016, the home dialysis use in patients aged <65 years declined sharply. This decrease can in part be explained by an increase in kidney transplantations. In incident patients above 65 years of age, the uptake of home dialysis remained stable, possibly explained by initiatives to promote home dialysis in the elderly. This study demonstrated that the home dialysis population has aged considerably, which was more pronounced than the ageing of the dialysis population in general.

Within a growing population with ESKD, sufficient resources to facilitate home dialysis must be offered to support this older patient population in their dialysis modality of choice.

## CONFLICT OF INTEREST

AB's work is supported by The Dutch Kidney Foundation and ZonMw Government Institution. AB, AES, AA and BJ are involved in the DOMESTICO study (Netherlands Trial Register identifier: NL6519). They report grants of the Dutch Kidney Foundation (grant no: A2D4P02) and ZonMw Government Institution (grant no: 843004116) for the conduct of this study. All other authors declared no conflicts of interest. The results presented in this paper have not been published previously, except in abstract form in the supplemental issue of Nephrology Dialysis Transplantation distributed at the 58th ERA‐EDTA congress, Berlin, 5‐7 of June 2021.

## AUTHOR CONTRIBUTIONS

AB, FI designed the research question. AB performed the statistical analyses and drafted the manuscript. TH, FI and BJ provided guidance on data analysis and interpretation. MH, AES and AA provided intellectual content of critical importance to the work described. All authors critically edited the manuscript and approved the final version of the manuscript.

## Supporting information

Supplementary Material

## Data Availability

The data that support the findings of this study are available from Dutch Renal Registry (RENINE), Nefrovisie Foundation. Restrictions apply to the availability of these data, which were used under licence for this study. Data will be shared on request to the corresponding author with permission of Dutch Renal Registry (RENINE), Nefrovisie Foundation.

## References

[1] Couser WG , Remuzzi G , Mendis S , Tonelli M . The contribution of chronic kidney disease to the global burden of major noncommunicable diseases. Kidney Int. 2011;80(12):1258‐1270. 21993585 10.1038/ki.2011.368

[2] Xie Y , Bowe B , Mokdad AH , et al. Analysis of the global burden of disease study highlights the global, regional, and national trends of chronic kidney disease epidemiology from 1990 to 2016. Kidney Int. 2018;94(3):567‐581. 30078514 10.1016/j.kint.2018.04.011

[3] ERA‐EDTA Registry : ERA‐EDTA registry annual report 2018. Amsterdam UMC, location AMC. Department of Medical Informatics, Amsterdam, the Netherlands, 2020.

[4] Bonenkamp AA , van Eck van der Sluijs A , Hoekstra T , et al. Health‐related quality of life in home dialysis patients compared to in‐center hemodialysis patients: a systematic review and meta‐analysis. Kidney Med. 2020;2(2):139‐154. 32734235 10.1016/j.xkme.2019.11.005 PMC7380444

[5] Dahlerus C , Quinn M , Messersmith E , et al. Patient perspectives on the choice of dialysis modality: results from the empowering patients on choices for renal replacement therapy (EPOCH‐RRT) study. Am J Kidney Dis. 2016;68(6):901‐910. 27337991 10.1053/j.ajkd.2016.05.010

[6] Mohnen SM , van Oosten MJM , Los J , et al. Healthcare costs of patients on different renal replacement modalities ‐ analysis of Dutch health insurance claims data. PLoS One. 2019;14(8):e0220800. 31415578 10.1371/journal.pone.0220800 PMC6695145

[7] van de Luijtgaarden MW , Noordzij M , Stel VS , et al. Effects of comorbid and demographic factors on dialysis modality choice and related patient survival in Europe. Nephrol Dial Transplant. 2011;26(9):2940‐2947. 21325351 10.1093/ndt/gfq845

[8] Cecka JM . Kidney transplantation from living unrelated donors. Annu Rev Med. 2000;51(1):393‐406. 10774472 10.1146/annurev.med.51.1.393

[9] Robinson BM , Akizawa T , Jager KJ , Kerr PG , Saran R , Pisoni RL . Factors affecting outcomes in patients reaching end‐stage kidney disease worldwide: differences in access to renal replacement therapy, modality use, and haemodialysis practices. Lancet. 2016;388(10041):294‐306. 27226132 10.1016/S0140-6736(16)30448-2 PMC6563337

[10] ERA‐EDTA Registry : ERA‐EDTA registry 2003 annual report. Academic Medical Center, Amsterdam, The Netherlands, 2005.

[11] Hoekstra T , Dekker FW , Cransberg K , Bos WJ , van Buren M , Hemmelder MH . RENINE annual report 2018. Available at: https://www.nefrovisie.nl/jaarrapportages/. Accessed January 04, 2021

[12] Simera I , Moher D , Hoey J , Schulz KF , Altman DG . A catalogue of reporting guidelines for health research. Eur J Clin Invest. 2010;40(1):35‐53. 20055895 10.1111/j.1365-2362.2009.02234.x

[13] Benchimol EI , Smeeth L , Guttmann A , et al. The reporting of studies conducted using observational routinely‐collected health data (RECORD) statement. PLoS Med. 2015;12(10):e1001885. 26440803 10.1371/journal.pmed.1001885 PMC4595218

[14] Ethier I , Cho Y , Hawley C , et al. Effect of patient‐ and center‐level characteristics on uptake of home dialysis in Australia and New Zealand: a multicenter registry analysis. Nephrol Dial Transplant. 2020;35(11):1938‐1949. 32031636 10.1093/ndt/gfaa002

[15] Hemke AC , Dekker FW , Bos WJ , Krediet RT , Heemskerk MB , Hoitsma AJ . Causes of decreased use of peritoneal dialysis as a kidney replacement therapy in the Netherlands. Ned Tijdschr Geneeskd. 2012;156(21):1–8. 22617065

[16] Ministerie van Volksgezondheid, Welzijn en Sport . Staatscourant van het Koninkrijk der Nederlanden, Staatscourant 1999, 126 page 5. https://zoek.officielebekendmakingen.nl/stcrt‐1999‐126‐p5‐SC19556.html

[17] Kramer A , Pippias M , Noordzij M ,, et al. The European renal association – European dialysis and transplant association (ERA‐EDTA) registry annual report 2016: a summary. Clin Kidney J. 2019;12(5):702‐720. 31583095 10.1093/ckj/sfz011 PMC6768305

[18] Fine JP , Gray RJ . A proportional hazards model for the subdistribution of a competing risk. J Am Stat Assoc. 1999;94(446):496‐509.

[19] Walker DR , Inglese GW , Sloand JA , Just PM . Dialysis facility and patient characteristics associated with utilization of home dialysis. Clin J Am Soc Nephrol. 2010;5(9):1649‐1654. 20634324 10.2215/CJN.00080110 PMC2974407

[20] Blake P . Why is the proportion of patients doing peritoneal dialysis declining in North America? Perit Dial Int. 2001;21(2):107‐116. 11330552

[21] Li PK‐T , Chow KM , Van de Luijtgaarden MWM , et al. Changes in the worldwide epidemiology of peritoneal dialysis. Nat Rev Nephrol. 2017;13(2):90‐103. 28029154 10.1038/nrneph.2016.181

[22] ANZDATA Registry . 43rd report, chapter 2: prevalence of renal replacement therapy for end stage kidney disease. Australia and New Zealand Dialysis and Transplant Registry, Adelaide, Australia. 2020. Available at: http://www.anzdata.org.au. Accessed July 13, 2021

[23] Leung CB , Cheung WL , Li PK . Renal registry in Hong Kong‐the first 20 years. Kidney Int Suppl. 2015;5(1):33‐38. 10.1038/kisup.2015.7 PMC4455190 26097783

[24] Johansen KL , Chertow GM , Foley RN , et al. US renal data system 2020 annual data report: epidemiology of kidney disease in the United States. Am J Kidney Dis. 2021;77(4 Supplement 1):A7‐A8. 33752804 10.1053/j.ajkd.2021.01.002 PMC8148988

[25] Sedor JR , Watnick S , Patel UD , et al. ASN end‐stage renal disease task force: perspective on prospective payments for renal dialysis facilities. J Am Soc Nephrol. 2010;21(8):1235‐1237. 20675502 10.1681/ASN.2010060656

[26] Honkanen EO , Rauta VM . What happened in Finland to increase home hemodialysis? Hemodial Int. 2008;12:S11‐S15. 18638234 10.1111/j.1542-4758.2008.00289.x

[27] van de Luijtgaarden MW , Jager KJ , Segelmark M , et al. Trends in dialysis modality choice and related patient survival in the ERA‐EDTA Registry over a 20‐year period. Nephrol Dial Transplant. 2016;31(1):120‐128. 26311215 10.1093/ndt/gfv295

[28] Boenink R , Stel VS , Waldum‐Grevbo BE , et al. Data from the ERA‐EDTA registry were examined for trends in excess mortality in European adults on kidney replacement therapy. Kidney Int. 2020;98(4):999‐1008. 32569654 10.1016/j.kint.2020.05.039

[29] Segall L , Nistor I , Van Biesen W , et al. Dialysis modality choice in elderly patients with end‐stage renal disease: a narrative review of the available evidence. Nephrol Dial Transplant. 2017;32(1):41‐49. 26673908 10.1093/ndt/gfv411

[30] Oliver MJ , Quinn RR , Richardson EP , Kiss AJ , Lamping DL , Manns BJ . Home care assistance and the utilization of peritoneal dialysis. Kidney Int. 2007;71(7):673‐678. 17264874 10.1038/sj.ki.5002107

[31] Hurst H , Figueiredo AE . The needs of older patients for peritoneal dialysis: training and support at home. Perit Dial Int. 2015;35(6):625‐629. 26702002 10.3747/pdi.2014.00337 PMC4689463

[32] Maaroufi A , Fafin C , Mougel S , et al. Patients' preferences regarding choice of end‐stage renal disease treatment options. Am J Nephrol. 2013;37(4):359‐369. 23548342 10.1159/000348822

[33] Brown EA , Johansson L . Dialysis options for end‐stage renal disease in older people. Nephron Clin Pract. 2011;119(Suppl 1):c10‐c13. 10.1159/000328019 21832850

[34] Iyasere OU , Brown EA , Johansson L , et al. Quality of life and physical function in older patients on dialysis: a comparison of assisted peritoneal dialysis with hemodialysis. Clin J Am Soc Nephrol. 2016;11(3):423‐430. 26712808 10.2215/CJN.01050115 PMC4785682

[35] Castledine CI , Gilg JA , Rogers C , Ben‐Shlomo Y , Caskey FJ . Renal centre characteristics and physician practice patterns associated with home dialysis use. Nephrol Dial Transplant. 2013;28(8):2169‐2180. 23737483 10.1093/ndt/gft196

[36] Rioux JP , Cheema H , Bargman JM , Watson D , Chan CT . Effect of an in‐hospital chronic kidney disease education program among patients with unplanned urgent‐start dialysis. Clin J Am Soc Nephrol. 2011;6(4):799‐804. 21212422 10.2215/CJN.07090810 PMC3069372

