# Supplementary material for: Trends in home dialysis use differ among age categories in past two decades: A Dutch registry study
Source: Eur J Clin Invest. 2021 Aug 2;52(1):e13656. doi: 10.1111/eci.13656 (PMC9286376; doi:10.1111/eci.13656)
Supplement: Supplementary file 1 — Supplementary Material [file ECI-52-0-s001.docx]

**Supplementary material for ‘Trends in home dialysis use differ among age categories in past two decades: a Dutch registry study’.**

Anna A Bonenkamp^1^, Tiny Hoekstra^1,2^, Marc H Hemmelder^2,3^, Anita van Eck van der Sluijs^4^, Alferso C Abrahams^4^, Frans J van Ittersum^1^ and Brigit C van Jaarsveld^1^*

*^1^* *Department of Nephrology, Amsterdam UMC, Vrije Universiteit Amsterdam, Research institute Amsterdam Cardiovascular Sciences, Amsterdam, the Netherlands*

*^2^Dutch Renal Registry (RENINE), Nefrovisie Foundation, Utrecht, the Netherlands.*

*^3^ Department of Nephrology, Medical University Center Maastricht, Maastricht, the Netherlands*

*^4^ Department of Nephrology and Hypertension, University Medical Center Utrecht, Utrecht, the Netherlands*

Corresponding author:
B.C. van Jaarsveld, MD, PhD

e-mail:b.jaarsveld@amsterdamumc.nl

**Supplementary Table S1.** Uptake of home haemodialysis within the first 2 years of dialysis initiation (n=33,340), by time period

|  | Time period | | | |
| --- | --- | --- | --- | --- |
|  | 1997-2001 OR (95% CI) | 2002-2006 ^a^ | 2007-2011 OR (95% CI) | 2012-2016 OR (95% CI) |
| *unadjusted*  *adjusted^b^* | 0.72 (0.50 – 1.02)  0.63 (0.43 – 0.93) | 1.0  1.0 | 1.26 (0.93 – 1.71)  1.36 (0.99 – 1.87) | 2.90  (2.17 – 3.88)  3.57 (2.59 – 4.92) |

^a^ time period 2002-2006 was regarded as reference period
*^b^* adjusted for sex, age, dialysis vintage, and transplantation history

**Supplementary Table S2.** Sensitivity analysis: uptake of home dialysis in the first 2 years of dialysis initiation in patients with a first dialysis episode in 1997-2016 (n=29,892), by time period and age category

|  |  | Time period | | | |
| --- | --- | --- | --- | --- | --- |
|  |  | 1997-2001  OR (95% CI) | 2002-2006 ^a^ | 2007-2011 OR (95% CI) | 2012-2016  OR (95% CI) |
| **Age 20-44 years** | *unadjusted*  *adjusted^b^* | 1.63  (1.37 – 1.93)  1.63 (1.37 – 1.93) | 1.0  1.0 | 0.71  (0.59 – 0.85)  0.70 (0.59 – 0.84) | 0.54  (0.45 – 0.66)  0.54 (0.45 – 0.66) |
| **Age 45-64 years** | *unadjusted*  *adjusted^b^* | 1.30  (1.17 – 1.46)  1.30 (1.17 – 1.46) | 1.0  1.0 | 0.80 (0.70 – 0.88)  0.79  (0.70 – 0.88) | 0.59 (0.52 – 0.66)  0.59 (0.52 – 0.66) |
| **Age 65-74 years** | *unadjusted*  *adjusted^b^* | 1.30 (1.13 – 1.49)  1.30 (1.14 – 1.50) | 1.0  1.0 | 0.96 (0.84 – 1.10)  0.96  (0.84 – 1.10) | 0.99 (0.87 – 1.13)  0.99  (0.86 – 1.13) |
| **Age above 75 years** | *unadjusted*  *adjusted^b^* | 1.35 (1.10 – 1.66)  1.36  (1.10 – 1.67) | 1.0  1.0 | 1.24 (1.05 – 1.48)  1.24  (1.04 – 1.48) | 1.55  (1.31 – 1.84)  1.54  (1.30 – 1.82) |

^a^ time period 2002-2006 was regarded as reference period
*^b^* adjusted for sex and transplantation history

**Supplementary Table S3.** Sensitivity analysis: uptake of home dialysis within the first 2 years of dialysis initiation in dialysis episodes with a dialysis duration of at least 2 years (n=20,665), by time period and age category

|  |  | Time period | | | |
| --- | --- | --- | --- | --- | --- |
|  |  | 1997-2001 OR (95% CI) | 2002-2006 ^a^ | 2007-2011 OR (95% CI) | 2012-2016 OR (95% CI) |
| **Age 20-44 years** | *unadjusted*  *adjusted^b^* | 2.05 (1.48 – 2.86)  1.96 (1.43 – 2.68) | 1.0  1.0 | 0.45 (0.31 – 0.64)  0.49 (0.35 – 0.68) | 0.37  (0.25 – 0.55)  0.42 (0.29 – 0.61) |
| **Age 45-64 years** | *unadjusted*  *adjusted^b^* | 1.72 (1.37 – 2.16)  1.71 (1.36 – 2.15) | 1.0  1.0 | 0.59 (0.47 – 0.74)  0.60 (0.48 – 0.75) | 0.40  (0.30 – 0.52)  0.41  (0.31 – 0.52) |
| **Age 65-74 years** | *unadjusted*  *adjusted^b^* | 1.42  (1.20 – 1.69)  1.42 (1.20 – 1.68) | 1.0  1.0 | 0.95  (0.81 – 1.13)  0.95  (0.81 – 1.12) | 0.88  (0.75 – 1.04)  0.88 (0.75 – 1.04) |
| **Age above 75 years** | *unadjusted*  *adjusted^b^* | 1.09  (0.82 – 1.45)  1.09 (0.82 – 1.45) | 1.0  1.0 | 1.16  (0.93 – 1.44)  1.16 (0.93 – 1.45) | 1.40 (1.13 – 1.73)  1.40 (1.13 – 1.73) |

^a^ time period 2002-2006 was regarded as reference period
*^b^* adjusted for sex, dialysis vintage, and transplantation history

**Appendix S1.** Complete list of codes used to classify study outcomes and events during follow-up

| **VALUE = therap** | **Original code in registry** | **Defined as** |
| --- | --- | --- |
|  | in-centre HD  Including active in-centre HD and nocturnal in-centre HD | CHD |
|  | Home HD | Home HD |
|  | PD  Including CAPD and APD | PD |
|  | Kidney transplantation  Including deceased donor kidney transplantation and living donor kidney transplantation | Kidney transplantation |
|  | Lost to follow-up | Lost to follow-up |
|  | Informed consent withdrawn | Lost to follow-up |
|  | Recovery of kidney function | Recovery of kidney function |
|  | Conservative treatment  *In use from 2015* | Lost to follow-up |
|  | Stop dialysis treatment and start palliative care | Death |
|  | Death | Death |
